# Exploration of the threshold SUV for diagnosis of malignancy using ^18^F-FBPA PET/CT

**DOI:** 10.1186/s41824-022-00156-z

**Published:** 2022-12-05

**Authors:** Kayako Isohashi, Yasukazu Kanai, Teruhito Aihara, Naonori Hu, Kentaro Fukushima, Ichiro Baba, Fumitoshi Hirokawa, Ryo Kakino, Tsuyoshi Komori, Keiji Nihei, Jun Hatazawa, Koji Ono

**Affiliations:** 1 Department of Kansai BNCT Medical Center, Osaka Medical and Pharmaceutical University, 2-7, Daigaku-Machi, Takatsuki City, Osaka, 569-8686 Japan; 2 Department of Nuclear Medicine and Tracer Kinetics, Osaka University Graduate School of Medicine, 2-2, Yamadaoka, Suita City, Osaka, 565-0871 Japan; 3 Department of Biofunctional Analysis, Osaka Medical and Pharmaceutical University, 4-20-1, Nasahara, Takatsuki City, Osaka, 569-1094 Japan; 4 Particle Radiation Oncology Research Center, Institute for Integrated Radiation and Nuclear Science, Kyoto University, 2 Asashiro Nishi, Kumatori Cho, Sennnan District, Osaka, 590-0494 Japan; 5 Department of Hematology and Oncology, Osaka University Graduate School of Medicine, Suita City,Osaka, Japan; 6 Department of Orthopedic Surgery, Osaka Medical and Pharmaceutical University, Takatsuki City, Osaka, Japan; 7 Department of General and Gastroenterological Surgery, Osaka Medical and Pharmaceutical University, Takatsuki City, Osaka, Japan

**Keywords:** FBPA, FDG, PET/CT, SUV, Malignancy, Benign

## Abstract

**Background:**

The goal of the study was to evaluate the diagnostic ability of ^18^F-FBPA PET/CT for malignant tumors. Findings from ^18^F-FBPA and ^18^F-FDG PET/CT were compared with pathological diagnoses in patients with malignant tumors or benign lesions.

**Methods:**

A total of 82 patients (45 males, 37 females; median age, 63 years; age range, 20–89 years) with various types of malignant tumors or benign lesions, such as inflammation and granulomas, were examined by ^18^F-FDG and ^18^F-FBPA PET/CT. Tumor uptake of FDG or FBPA was quantified using the maximum standardized uptake value (SUVmax). The final diagnosis was confirmed by cytopathology or histopathological findings of the specimen after biopsy or surgery. A ROC curve was constructed from the SUVmax values of each PET image, and the area under the curve (AUC) and cutoff values were calculated.

**Results:**

The SUVmax for ^18^F-FDG PET/CT did not differ significantly for malignant tumors and benign lesions (10.9 ± 6.3 vs. 9.1 ± 2.7 *P* = 0.62), whereas SUVmax for ^18^F-FBPA PET/CT was significantly higher for malignant tumors (5.1 ± 3.0 vs. 2.9 ± 0.6, *P* < 0.001). The best SUVmax cutoffs for distinguishing malignant tumors from benign lesions were 11.16 for ^18^F-FDG PET/CT (sensitivity 0.909, specificity 0.390) and 3.24 for ^18^F-FBPA PET/CT (sensitivity 0.818, specificity 0.753). ROC analysis showed significantly different AUC values for ^18^F-FDG and ^18^F-FBPA PET/CT (0.547 vs. 0.834, *p* < 0.001).

**Conclusion:**

^18^F-FBPA PET/CT showed superior diagnostic ability over ^18^F-FDG PET/CT in differential diagnosis of malignant tumors and benign lesions. The results of this study suggest that ^18^F-FBPA PET/CT diagnosis may reduce false-positive ^18^F-FDG PET/CT diagnoses.

## Background

Malignant tumors require prompt and accurate diagnosis due to the severity of the disease. If a malignant tumor is suspected on morphological imaging using X-ray, US, CT, and MRI, but these examinations do not provide a definitive diagnosis, functional imaging with PET/CT is performed. In particular, ^18^F-fluorodeoxyglucose (FDG) PET/CT is used to examine the stage, therapeutic effect, and recurrence of malignant tumors by utilizing the property that malignant tumor cells take up 3–8 times more glucose than normal cells (Yamada et al. 1995). However, inflammatory cells also take up glucose strongly, and thus, ^18^F-FDG PET/CT can also detect inflammatory activity (Kubota et al. 1992). In fact, in cardiac sarcoidosis and large vasculitis such as aortitis syndrome and giant cell arteritis, ^18^F-FDG PET/CT is performed in cases in which it is difficult to evaluate the lesion activity (Tang et al. 2016; Geest et al. 2021). Due to these properties, it is often difficult to distinguish malignant tumors from inflammatory lesions on ^18^F-FDG PET/CT. In such cases, invasive techniques such as biopsy and surgery are required for definitive diagnosis.

^18^F-FBPA PET/CT uses ^18^F-labeled 2-borono-4-fluoro-L-phenylalanine (^18^F-FBPA), rather than ^18^F-FDG, as a marker. ^18^F-FBPA is a boronate compound of phenylalanine that has behavior similar to that of natural phenylalanine in cells, and thus, its uptake reflects amino acid metabolism (Ishiwata 2019). This metabolism is increased in malignant tumor cells, and ^18^F-FBPA selectively accumulates in malignant tumors (Watabe et al. 2017; Hanaoka et al. 2014), whereas physiological accumulation of ^18^F-FBPA is low in normal organs, except for the urinary system (Shimosegawa et al. 2016; Romanov et al. 2020). ^18^F-FBPA-PET has been used in clinical studies for assessment of tumor uptake of boron (^10^B) during boron neutron capture therapy (BNCT) for refractory and recurrent head and neck cancer and brain tumors (Imahori et al. 1998; Kato et al. 2009). In Japan, BNCT for refractory and recurrent head and neck cancer has been covered by health insurance since June 2020, and the number of diseases insured for treatment with BNCT is likely to increase in the future.^18^F-FBPA PET/CT has also been reported to be useful for differentiating brain tumor recurrence from post-treatment changes such as radiation necrosis and pseudoprogression, but there are no definitive reports on use of this method for tumors and inflammatory lesions in the trunk (Beshr et al. 2018). Therefore, in this study, the diagnostic abilities of ^18^F-FBPA and ^18^F-FDG PET/CT were examined in cases with malignancy or inflammation throughout the body, based on comparison with pathological diagnoses.

## Methods

### Selection of patients

All patients were referred for experimental diagnostics by treating oncologists facing an unmet diagnostic challenge that could not be solved with standard approaches. This prospective study was approved by the Clinical Research Ethics Committee of the Osaka Medical and Pharmaceutical University (CRB 19-01) and conducted in accordance with the 1964 Declaration of Helsinki and its later amendments or comparable ethical standards. Patients were recruited for the study from March 2020 through March 2022 with agreement from oncologists and after determination of eligibility.

The inclusion criteria were: (i) ^18^F-FDG PET/CT findings suspicious for primary and/or metastatic lesions and/or benign lesions such as inflammation or granuloma with abnormal ^18^F-FDG uptake; (ii) suspected or newly diagnosed or previously treated malignancies and/or benign lesions; (iii) age ≥ 20 years old; (iv) agreement to undergo biopsy; (v) provision of informed consent according to the guidelines of the Clinical Research Ethics Committee. The exclusion criteria were: (i) pregnant women or those who wish to become pregnant; and (ii) treatment started before PET/CT was performed.

A total of 82 patients (45 males, 37 females; median age, 63 years; age range, 20–89 years) with various types of malignant tumors and benign lesions were examined in 87 ^18^F-FBPA PET/CT and ^18^F-FDG PET/CT studies from March 2020 through March 2022. These patients included five with recurrence after treatment who underwent repeated PET examinations. The final definitive diagnosis was confirmed by cytopathological or histopathological findings of the specimen after biopsy or resection.

### Preparation of ^18^F-FDG and ^18^F-FBPA

^18^F-FDG was produced using the standard method in our laboratory. ^18^F-FBPA was synthesized as previously described (Ishiwata et al. 1991; Isohashi et al. 2016). A MSP-200 synthesizer (Sumitomo Heavy Industries, Tokyo, Japan) was used, with 4-borono L-phenylalanine (Sigma-Aldrich, St. Louis, USA or STELLA PHARMA CORPORATION, Osaka, Japan) as the precursor. Purification of ^18^F-FBPA was performed by high-performance liquid chromatography (HPLC) using a YMC-Pack ODS-A column (250 × 150 mm; YMC, Kyoto, Japan) eluted with 0.1% acetic acid at a flow rate of 10 mL/min. The radiochemical purity was > 95% for ^18^F-FDG and ^18^F-FBPA, and the final product was sterile and pyrogen-free.

### PET/CT imaging

The median interval between ^18^F-FDG and ^18^F-FBPA PET/CT scans was 5 days (range, 1–72 days). The ^18^F-FDG PET/CT scan was performed first. Patients fasted for at least 4 h before ^18^F-FDG PET/CT scans to ensure a normal glucose level in the peripheral blood. ^18^F-FBPA PET/CT was also performed in patients who had fasted for at least 4 h to align the conditions with ^18^F-FDG PET/CT. The dose of intravenously injected ^18^F-FDG or ^18^F-FBPA was calculated based on body weight (3.7–5.0 MBq/kg for ^18^F-FDG and ^18^F-FBPA) (Beshr et al. 2018). Patients were asked to void their bladders before each scan. Data were acquired using a hybrid PET/CT scanner (Discovery PET/CT 710, GE Healthcare, Milwaukee, WI, USA) after intravenous administration for 60 ± 10 min. The imaging range was from the top of the head to the thigh or to the toe for lesions in the lower extremities.

CT was performed with tube voltage 120 kV, current 100 mA and slice thickness 3.75 mm. A PET scan was immediately performed after the CT scan in 3D acquisition mode with 6–8 bed positions and 2.0 min/position. Data were transferred to an Advantage Workstation (AW 2.0, GE Healthcare) and reconstructed using the ordered subset expectation maximization algorithm (two iterations and 18 subsets) using CT data for attenuation correction. The reconstructed images were then co-registered and displayed. PET images were analyzed qualitatively (presence or absence of tracer uptake outside sites of physiological accumulation or excretion) and semi-quantitatively using a volumetric volume of interest (VOI) placed over the target lesion and tailored to the extent of each lesion. The maximum tumor standardized uptake value (SUVmax) for each VOI was automatically generated by the tomography software. Standard vital signs (blood pressure, heart rate and body temperature) were checked between ^18^F-FDG and ^18^F-FBPA injections and up to 120 min after completion of the PET/CT scan, and patients were asked to report any abnormalities.

### Target lesion uptake of ^18^F-FDG and ^18^F-FBPA

Lesion uptake of ^18^F-FDG and ^18^F-FBPA on PET images and quantification of the SUV were performed using AW Volume Share software (GE Healthcare). VOIs were delineated on axial 3-D images. The SUV was defined as regional radioactivity divided by injected radioactivity normalized to body weight. ^18^F-FDG and ^18^F-FBPA uptake were evaluated using SUVmax at 1 h after injection (Igaki et al. 2020).

### Statistical analysis

Statistical analyses were performed using R software (ver. 4.0.3) and Excel. Results for ^18^F-FDG and ^18^F-FBPA PET/CT were compared with histopathological findings. The difference between the mean SUVmax for malignant tumors and benign lesions for each PET method was evaluated by Mann–Whitney U test. The sensitivity, specificity, positive predictive value (PPV), negative predictive value (NPV) and accuracy of ^18^F-FDG and ^18^F-FBPA PET/CT were calculated and compared to evaluate the diagnostic efficacy.

Receiver-operating characteristic (ROC) curves were generated from the SUVmax of the target lesion for each PET method, and the area under the curve (AUC) and the best SUVmax cutoff for differentiating malignant tumors and benign lesions were obtained to give optimal sensitivity and specificity. The AUCs of the two markers for malignant tumors and benign lesions were compared statistically to evaluate the diagnostic performance of each PET method. Two-tailed *P* values < 0.05 were considered significant in all analyses.

## Results

### Adverse events

All patients tolerated ^18^F-FDG and ^18^F-FBPA PET/CT well. There were no signs of any drug-related pharmacologic effects or physiological responses. All observed vital signs (including blood pressure, heart rate, and body temperature) remained within normal limits during and after ^18^F-FBPA PET/CT. None of the patients reported any abnormal symptoms.

### ^18^F-FDG and ^18^F-FBPA uptake in tumors and benign lesions

**Fig. 1:**
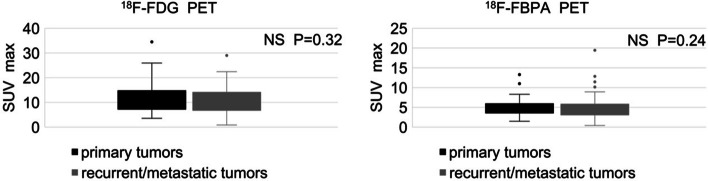
SUVmax of ^18^F-FDG and ^18^F-FBPA PET/CT for primary tumors vs. recurrent or metastatic tumors

**Fig. 2:**
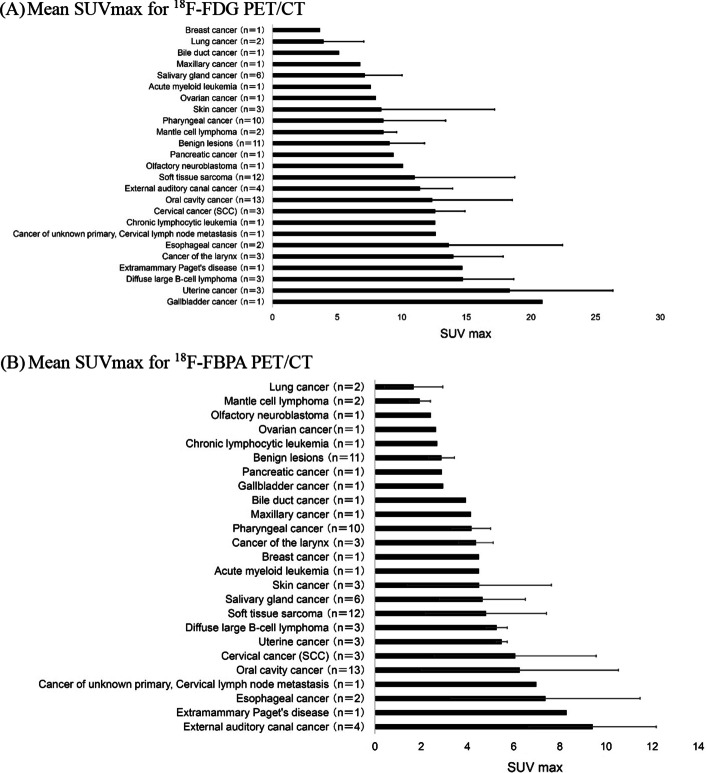
Mean SUVmax for (**A**) ^18^F-FDG and (**B**) ^18^F-FBPA PET/CT. Means and standard deviation for all lesions (n = 88) are shown by disease.* SCC* squamous cell carcinoma

Patient numbers were insufficient to compare the SUVs of primary tumors vs. recurrent or metastatic tumors for individual cancers. The details are summarized in Table 1. The overall mean SUVmax for primary tumors (*n* = 22) and recurrent/metastatic tumors (*n* = 55) did not differ significantly for ^18^F-FDG (12.3 ± 7.3 vs. 10.3 ± 5.7, *p* = 0.32) or ^18^F-FBPA (5.3 ± 2.6 vs. 5.0 ± 3.1, *p* = 0.24) (Fig. 1). Subsequently, primary and recurrent/metastatic tumors were analyzed in a pooled fashion. Most malignant tumors were highly sensitive to ^18^F-FDG uptake, with a mean SUVmax ≥ 5 (Fig. 2A). The mean SUVmax of benign lesions was > 9, also indicating strong ^18^F-FDG uptake. The highest mean SUVmax (> 9) on ^18^F-FBPA PET/CT occurred for external auditory canal cancer (Fig. 2B), whereas low ^18^F-FBPA uptake (mean SUVmax < 3) was found in well-differentiated lung cancer, mantle cell lymphoma, olfactory neuroblastoma, ovarian cancer, chronic lymphocytic leukemia, pancreatic cancer, gallbladder cancer and benign lesions.

**Table 1:** Patient characteristics

Total no. of patients	82
Male/Female	45/37
Median age (range)	63 (range 20–89 years)
Total no. of each PET study with ^18^F-FDG and ^18^F-FBPA	87
Total no. of target lesions with pathological diagnosis	88
Malignant tumor (primary/recurrent or metastatic)	77 (22/55)
Oral cavity cancer	13
Soft tissue sarcoma	12
Pharyngeal cancer	10
Salivary gland cancer	6
External auditory canal cancer	4
Cancer of the larynx	3
Skin cancer (mucinous/SCC)	3 (2/1)
Diffuse large B-cell lymphoma	3
Uterine cancer (endometrial cancer)	3
Cervical cancer of Uterus (SCC)	3
Lung cancer (adenocarcinoma)	2
Mantle cell lymphoma	2
Esophageal cancer	2
Olfactory neuroblastoma	1
Ovarian cancer	1
Chronic lymphocytic leukemia	1
Pancreatic cancer	1
Gallbladder cancer	1
Bile duct cancer	1
Maxillary cancer	1
Breast cancer	1
Acute myeloid leukemia	1
Cancer of unknown primary, Cervical lymph node metastasis	1
Extramammary Paget’s disease	1
Benign lesion	11
Inflammatory granulation	2
Radiation osteomyelitis	2
Abscess	2
Inflammatory lymphadenopathy	1
Tonsillitis	1
Post-treatment change after chemoradiotherapy	1
Cholecystitis	1
Graft versus host disease	1

*SCC* squamous cell carcinoma

### Detection of target lesions

**Fig. 3:**
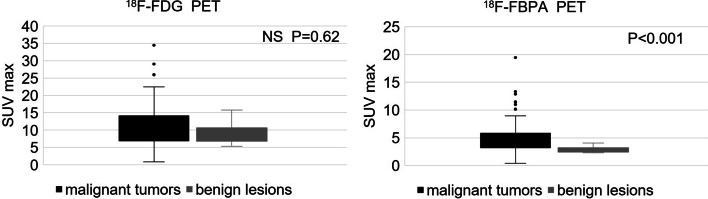
SUVmax of ^18^F-FDG and ^18^F-FBPA PET/CT for malignant tumors vs. benign lesions

The final histopathologic results showed 77 lesions with 24 types of malignant tumors and 11 benign lesions. SUVmax on ^18^F-FDG PET/CT did not differ significantly for malignant tumors and benign lesions (10.9 ± 6.3 vs. 9.1 ± 2.7, *P* = 0.62). In contrast, SUVmax on ^18^F-FBPA PET/CT was significantly higher for malignant tumors compared to benign lesions (5.1 ± 3.0 vs. 2.9 ± 0.6, *P* < 0.001) (Fig. 3).

**Fig. 4:**
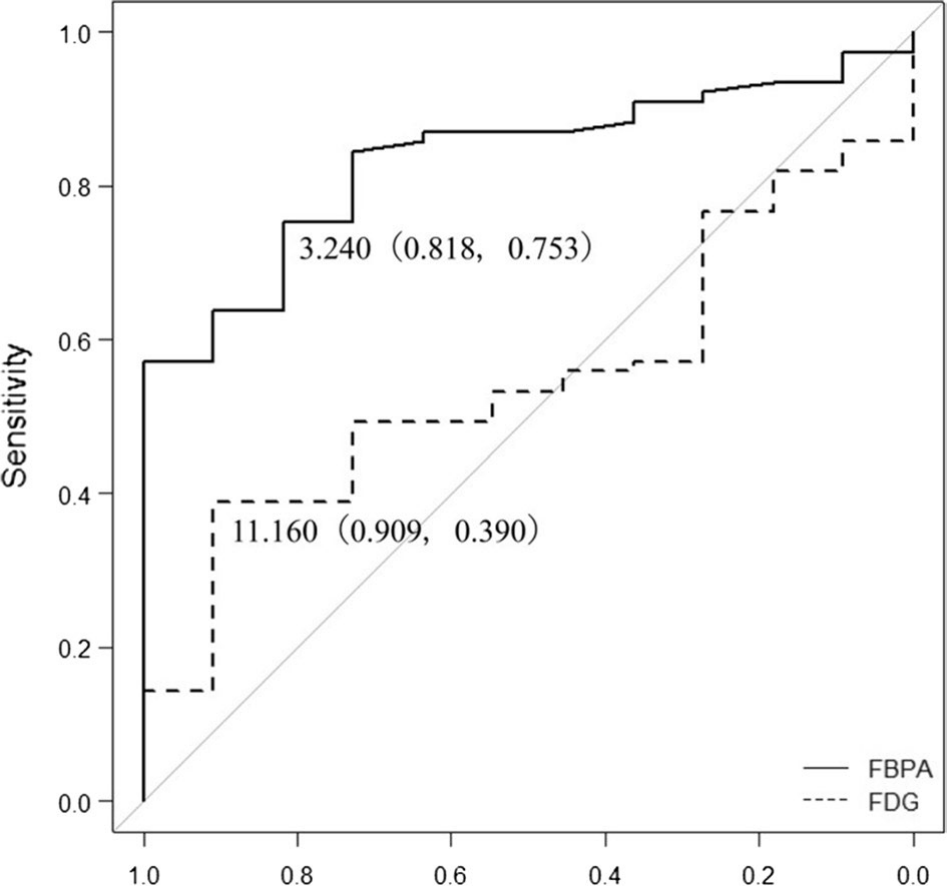
ROC curves for SUVmax on ^18^F-FDG (broken line) and ^18^F-FBPA (solid line) PET/CT

**Fig. 5:**
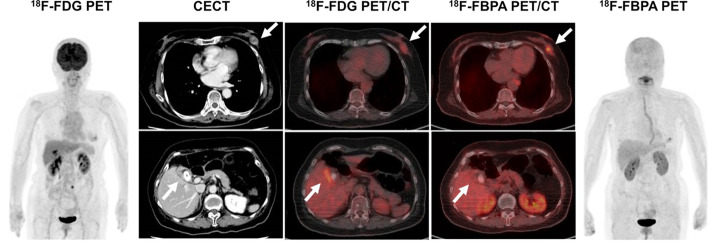
An 80-year-old woman underwent contrast-enhanced CT (CECT) for a recently diagnosed breast tumor before surgery. CECT revealed gallbladder wall thickening with gallstones and a contrast enhancement effect, in addition to a left mammary mass, which may indicate gallbladder cancer. ^18^F-FDG PET/CT showed a local increase in accumulation in the gallbladder wall (SUVmax: 8.1), making it difficult to differentiate gallbladder cancer from cholecystitis. ^18^F-FBPA PET/CT showed accumulation in the left breast mass (SUVmax: 4.5), but no significant accumulation in the gallbladder wall (SUVmax: 2.8). Cholecystectomy was performed and the pathological diagnosis was inflammatory granulation tissue

**Fig. 6:**
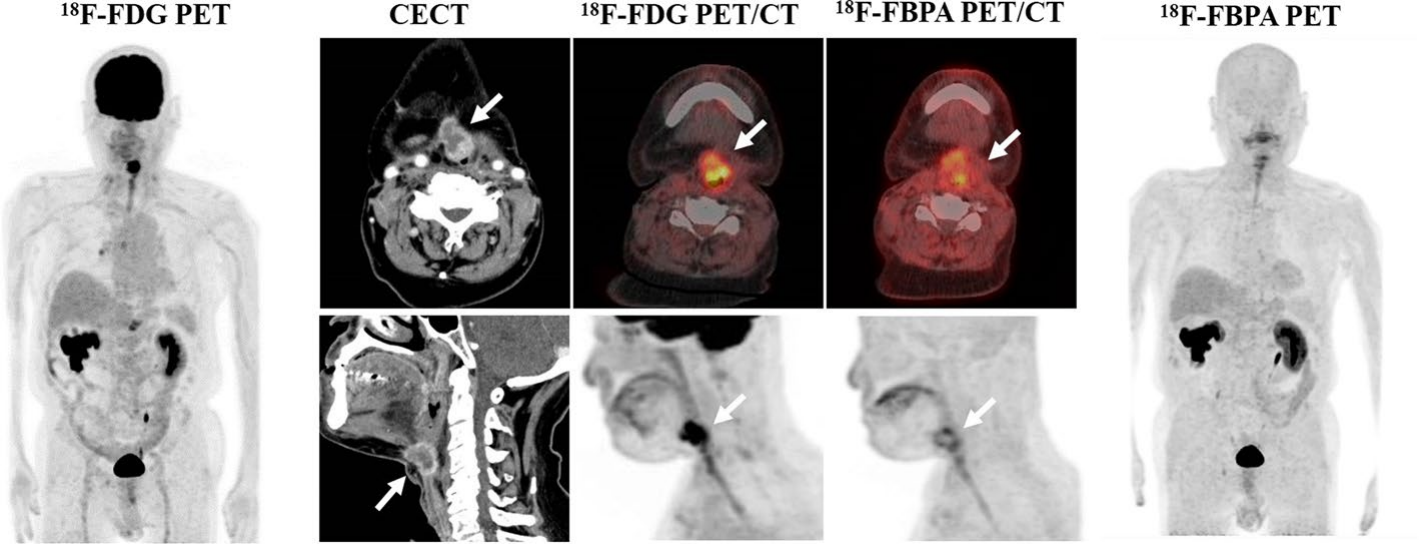
A 75-year-old man underwent contrast-enhanced CT to confirm local recurrence of hypopharyngeal cancer after radiotherapy. Clinically, post-treatment changes (granulation tissue, radiation necrosis) were suspected, but both ^18^F-FDG (SUVmax: 13.0) and ^18^F-FBPA (SUVmax: 5.8) PET/CT showed increased accumulation. Local recurrence was confirmed pathologically by puncture cytology

In a lesion-based analysis, the sensitivity, specificity, PPV, NPV and accuracy of the diagnosis were 98.7, 22.2, 87.4, 66.7 and 86.4% for ^18^F-FDG PET/CT and 94.8, 72.7, 96.0, 66.7 and 92% for ^18^F-FBPA PET/CT, respectively (Table 2). Thus, ^18^F-FDG PET/CT gave higher sensitivity, but lower specificity, PPV and accuracy compared to those of ^18^F-FBPA PET/CT. In ROC analysis (Fig. 4) of differential diagnosis of malignant and benign lesions, AUCs differed significantly for ^18^F-FDG and ^18^F-FBPA PET/CT (0.55 vs. 0.83,* P* < 0.001). The respective SUVmax cutoffs were 11.16 and 3.24. Representative cases are shown in Figs. 5 and 6.

**Table 2:** Diagnostic performance of ^18^F-FDG and ^18^F-FBPA PET/CT in target lesions

Modality	Sensitivity (%)	Specificity (%)	PPV (%)	NPV (%)	Accuracy (%)
^18^F-FDG PET/CT	98.7	22.2	87.4	66.7	86.4
^18^F-FBPA PET/CT	94.8	72.7	96	66.7	92

## Discussion

Uptake of amino acids and glucose in cancer cells is elevated to maintain rapid cell proliferation and intracellular metabolism (Oda 2014). Increased amino acid metabolism is mediated by elevated activity and expression of transporters, which are responsible for cellular uptake of nutrients (Kanai 2022), while glucose is transported into normal cells and cancer cells via the glucose transporter (GLUT) 1 (Kozal et al. 2021). Glucose metabolism is accelerated in malignant tumors, and PET uses a radioactive agent to visualize the status of metabolism (Hatazawa et al. 2003). ^18^F-FDG is a substrate for GLUT1 and is taken up by normal tissues such as the brain, resulting in high background values. Therefore, ^18^F-FDG PET has limited utility in diagnosis of malignant brain tumors (Kinoshita et al. 2012; Furuse et al. 2019). Granulation tissue, macrophages, and neutrophils also have elevated glucose metabolism, so high uptake of ^18^F-FDG occurs in inflammatory lesions in which these cells are proliferating. Kubota et al. found higher uptake of ^18^F-FDG in macrophages around necrotic tissue and in juvenile granulation tissue around tumors, rather than in the tumor cells themselves (Kubota et al. 1994). Thus, ^18^F-FDG PET/CT is limited for differential diagnosis of tumors and other lesions, especially in cases with active inflammation. The present results showed no significant difference in SUVmax of ^18^F-FDG PET/CT for malignant tumors and benign lesions (10.9 ± 6.3 vs. 9.1 ± 2.7, *P* = 0.62), indicating that differentiation is difficult, as also found in previous reports (Kratochwil et al. 2019; Bertagna et al. 2015; Plaxton et al. 2015).

The amino acid transport system includes consists of various amino acid transporters.

Recently, LAT1 of the L-type family of amino acid transporters has been shown to be present in tumor cells, while LAT2 is found in normal cells (Khunweeraphong et al. 2012). LAT1 is upregulated in many cancer cells and is highly correlated with the cell proliferation index, disease stage and poor prognosis (Lu et al. 2020; Kaira et al. 2009, 2012, 2013; Isoda et al. 2014; Shimizu et al. 2015; Yoshimoto et al. 2013). However, LAT1 is also expressed, although to a lesser extent, in cells and tissues with high proliferative and differentiation potential, such as the normal blood–brain barrier, blood-retina-brain barrier, placental barrier, endocrine glands and activated T cells (Wiriyasermkul et al. 2021). LAT2 is a neutral amino acid transporter that is mainly responsible for amino acid transport in the small intestine, where nutrients are absorbed, and in mucosal epithelial cells of the kidney, where amino acids are reabsorbed from urine (Khunweeraphong et al. 2012).

There are several amino acid PET tracers in clinical use, including ^11^C-MET (methionine), ^18^F-FAMT (methyltyrosine) and ^18^F-FBPA. ^11^C-MET is taken up by multiple amino acid transporters in normal cells since it is a natural amino acid used for protein synthesis in the cell, which leads to high background values in the liver, pancreas, and salivary gland tissue on PET (Wei et al. 2016; Isohashi et al. 2013). In contrast, ^18^F-FAMT is specific for LAT1 and is not transported by LAT2 (Wei et al. 2016). Results from ^18^F-FAMT PET in patients with lung cancer showed accumulation of ^18^F- FAMT in cancer foci that correlated with the LAT1 expression level, while normal tissue, inflammatory sites and benign lesions showed little accumulation of ^18^F-FAMT (Kaira et al. 2007). ^18^F-FAMT PET is superior to ^18^F-FDG PET for detection of malignant tumors in some cancer types (Inoue et al. 2001; Achmad et al. 2017). Kim et al. found that the SUV of ^18^F-FAMT is smaller than that of ^18^F-FDG (Kim et al. 2015) because ^18^F-FDG is also taken up by tumor-associated inflammation, whereas ^18^F-FAMT is not. Although ^18^F-FAMT is a promising tracer with higher specificity for cancer diagnosis than ^18^F-FDG, the limited amount of synthesis made it difficult to use in the clinical setting. Development of a simple and efficient method for ^18^F-FAMT is needed for clinical application (Inoue et al. 2001; Achmad et al. 2017).

BPA used in boron neutron capture therapy (BNCT) is transported by LAT1, LAT2 and another transporter of neutral amino acids, amino acid transporter B0 (ATB0). BPA is not specific for LAT1, but is mainly transported into cancer cells via LAT1 (Wongthai et al. 2015). In contrast, ^18^F-FBPA used in PET has been reported to be highly specific for LAT1 (Wongthai et al. 2015) and has been found to be useful in differential diagnosis of tumors and inflammation in animal models (Watabe et al. 2017). In the current study, ^18^F-FBPA uptake was visualized in many tumors, although ^18^F-FBPA tended not to show as high an uptake as ^18^F-FDG. A few tumors showed stronger accumulation of ^18^F-FBPA than ^18^F-FDG (SUVmax: external auditory canal cancer 13.3 vs. 11.6, breast cancer 4.5 vs. 3.7), but others showed low ^18^F-FBPA accumulation (SUVmax: mantle lymphoma 1.5–2.4, olfactory neuroblastoma 2.4) and could not be visualized (false-negative on PET). Conversely, a small number of cases of granulomatous inflammatory disease in the brainstem and cervical lymph node areas gave false-positive results. In such cases, it is important to evaluate the results in conjunction with other examinations and the clinical course. The expression level of LATI was not examined in this study, but is likely to vary among types of tumor, and this may also have caused differences in accumulation of ^18^F-FBPA. Well-differentiated lung cancers with frosted appearances on CT and ^18^F-FDG PET have low accumulation and cannot be visualized. This suggests that tumor size, cell density, and the limited spatial resolution of PET can also affect the diagnostic performance.

^18^F-FDG PET/CT has the advantage of capturing metabolic changes that precede morphological changes caused by therapeutic interventions, allowing early assessment of the effect of treatment. In malignant lymphoma, ^18^F-FDG PET/CT has been used to determine the response to treatment and to diagnose residual active disease (Isohashi et al. 2008). However, since ^18^F-FDG is also taken up by inflammatory lesions, it is limited for differentiating tumor and inflammatory responses to treatment, especially in patients with persistent inflammation (Miyashita et al. 2008). The present study suggests that this distinction may be possible under certain conditions, especially in the presence of inflammation after radiotherapy (Fig. 6).

Fibroblast activation protein (FAP) is a type II membrane-bound glycoprotein belonging to the dipeptidylpeptidase 4 family and is highly expressed in many epithelial cancer-associated fibroblasts. It is characterized by a strong desmoplastic response, and the association between FAP overexpression and a poor cancer prognosis has led to development of FAP-specific inhibitors (FAPIs) (Kratochwil et al. 2019). In recent years, FAPIs labeled with ^67^Ga- or ^18^F and detected by PET have been used to provide information on tumor diagnosis and radiotherapy planning (Chen et al. 2020; Fu et al. 2022). However, this technique (which is referred to as tumor stromal imaging) is intended to detect pathological conditions associated with tumors and does not directly depict the tumor cells themselves. In contrast, ^18^F-FBPA PET directly depicts tumor cells and has a different target dimension. In the context of treatment, ^18^F-FBPA PET allows for appropriate selection of patients for BNCT and accurate treatment planning (Aihara et al. 2020). In particular, prediction of boron concentrations in tumors is needed for accurate dose prediction and efficacy assessment in BNCT. Therefore, use of ^18^F- FBPA PET is important for further development of BNCT (Ono et al. 2019), and in this sense, the value of ^18^F-FBPA PET is extremely high. In this study, we mainly examined tumors and inflammation in the trunk region. There were some false-negative and false-positive cases, but SUVmax for ^18^F-FBPA PET differed significantly in tumors and inflammatory lesions, indicating sufficient discriminatory ability for clinical use. PET with amino acid tracers such as ^11^C-MET and ^18^F-FAMT can also help in differential diagnosis of tumors and inflammatory lesions, but ^18^F-FBPA PET has the added advantage of providing information relevant to BNCT treatment (Kim et al. 2015; Wedman et al. 2019). Currently, BNCT is performed for recurrent/refractory malignancies, but the threshold SUV of ^18^F-FBPA PET/CT diagnosis provides clues to differentiate malignancy from inflammatory lesions, including changes after radiotherapy, and may reduce the need for invasive biopsy and/or resection for pathological definitive diagnosis.

## Conclusion

The results of this study showed that ^18^F-FBPA PET/CT was superior to ^18^F-FDG PET/CT for differential diagnosis of malignant tumors and benign lesions. There are some limitations due to low ^18^F-FBPA accumulation in certain tumors, but the additional information obtained from ^18^F-FBPA PET/CT can reduce false positives from ^18^F-FDG PET/CT in tumor diagnosis.

## Data Availability

The datasets used and/or analyzed during the current study are available from the corresponding author on reasonable request.

## Abbreviations

^18^F-FDG: ^18^F-fluorodeoxyglucose
^18^F-FBPA: ^18^F-labeled 2-borono-4-fluoro-L-phenylalanine
SUVmax: Maximum tumor-standardized uptake value
